# Supplementary material for: Mycobacterium leprae infection, Hansen’s disease, and helminth infections: A cross-sectional study in southeastern Brazil
Source: medRxiv. 2025 Jun 27:2025.06.26.25330341. Preprint. [Version 1] doi: 10.1101/2025.06.26.25330341 (PMC12262754; doi:10.1101/2025.06.26.25330341)
Supplement: 1 [file NIHPP2025.06.26.25330341V1-supplement-1.pdf]

medRxiv preprint doi: https://doi.org/10.1101/2025.06.26.25330341; this version posted June 27, 2025. The copyright holder for this preprint (which was not certified by peer review) is the author/funder, who has granted medRxiv a license to display the preprint in perpetuity.
It is made available under a CC-BY 4.0 International license .

SUPPLEMENTARY TABLES AND FIGURES

Supplementary Table 1: Multivariate Logistic Regression with POSITIVE ANTI-LID1 and HISTORY OF HANSEN'S DISEASE against co-infection (serological results and *past parasitic infection*) and demographic risk factors

	**Pos Anti-LID-1**	**History of HD**
	aOR (95% CI)	aOR (95% CI)
S. mansoni antibody positive (anti-SEA)	0.63 (0.28, 1.41)	1.05 (0.50, 2.21)
S. sterocralis antibody positive (anti-NIE)	1.64 (0.72, 3.74)	0.45 (0.11, 1.94)
Past parasitic infection	0.97 (0.60, 1.57)	1.50 (0.80, 2.83)
Race (non-white)	1.15 (0.65, 2.02)	
Adult age (15 or older)		6.31 (0.85, 46.65)
Sex as female		0.64 (0.39, 1.07)
Rural residence	**1.86* (1.12, 3.07)**	**1.84* (1.06, 3.17)**

medRxiv preprint doi: https://doi.org/10.1101/2025.06.26.25330341; this version posted June 27, 2025. The copyright holder for this preprint (which was not certified by peer review) is the author/funder, who has granted medRxiv a license to display the preprint in perpetuity.
It is made available under a CC-BY 4.0 International license .

===

Note: Adjusted odds ratio listed with confidence intervals (in parentheses) of exposures involving positive serological results of helminth infections, reporting 'yes' to past parasitic infection, and best fit demographic variables. *p<0.05

Supplementary Table 2: MLR with <u>POSITIVE ANTI-LID1</u> and <u>HISTORY OF HANSEN'S DISEASE</u> against co-infection (serological results and *all individual parasites*) and demographic risk factors

===

	**Pos Anti-LID-1**	**History of HD**
	aOR (95% CI)	aOR (95% CI)
S. mansoni antibody positive (anti-SEA)	0.63 (0.28, 1.40)	1.09 (0.52, 2.31)
S. sterocralis antibody positive (anti-NIE)	1.63 (0.71, 3.76)	0.41 (0.09, 1.82)
Self-reported roundworm	0.97 (0.61, 1.53)	1.50 (0.90, 2.51)
Self-reported schistosomiasis	0.89 (0.52, 1.51)	0.92 (0.52, 1.63)
Self-reported hookworm	3.46 (0.82, 14.58)	0.79 (0.09, 6.82)
Self-reported tapeworm	1.88 (0.86, 4.11)	1.36 (0.54, 3.44)
Self-reported pinworm	0.58 (0.24, 1.45)	1.15 (0.46, 2.87)
Self-reported giardia	1.00 (0.34, 2.95)	1.39 (0.47, 4.16)
Self-reported amoeba	0.63 (0.26, 1.52)	0.49 (0.17, 1.41)
Self-reported other	0 (0, Inf)	3.51 (0.39, 33.45)
Race (non-white)	1.15 (0.69, 2.03)	

medRxiv preprint doi: https://doi.org/10.1101/2025.06.26.25330341; this version posted June 27, 2025. The copyright holder for this preprint (which was not certified by peer review) is the author/funder, who has granted medRxiv a license to display the preprint in perpetuity.
It is made available under a CC-BY 4.0 International license .

Adult age (15 or older)		6.85 (0.92, 50.85)
Sex as female		0.64 (0.378, 1.07)
Rural residence	**1.75* (1.03, 2.96)**	**1.84* (1.05, 3.25)**

Note: Adjusted odds ratio listed with confidence intervals (in parentheses) of exposures involving positive serological results of helminth infections, self-reported past or present infections, and best fit demographic variables. * p<0.05

Supplementary Table 3: Checking effects size change among different model permutations for the outcome of POSITIVE ANTI-LID1 (aOR)

	**Both A & SR**	**A Only**	**SR Only**
S. mansoni antibody positive (anti-SEA)	0.78		0.78
S. sterocralis antibody positive (anti-NIE)	1.57	1.54	1.57
Self-reported schistosomiasis	1.00	0.99	
Self reported helminth infection (other then S. mansoni)	1.14	1.15	1.14
Self-reported protozoa	0.96	0.95	0.96
Race (non-white)	1.21	1.21	1.21
Rural Residence	1.81**	1.79**	1.81**

Note: 'A' stands for antibody result for schistosomiasis. 'SR' is self report for schistomiasis. **p<0.05.

Supplementary Table 4: Checking effects size change among different model permutations for the outcome of HISTORY OF HANSEN'S DISEASE (aOR)

	**Both A & SR**	**A Only**	**SR Only**
S. mansoni antibody positive (anti-SEA)	1.25	1.25	
S. sterocralis antibody positive (anti-NIE)	0.40	0.40	0.40

medRxiv preprint doi: https://doi.org/10.1101/2025.06.26.25330341; this version posted June 27, 2025. The copyright holder for this preprint (which was not certified by peer review) is the author/funder, who has granted medRxiv a license to display the preprint in perpetuity.
It is made available under a CC-BY 4.0 International license .

Self-reported schistosomiasis	0.87		0.87
Self reported helminth infection (other then S. mansoni)	1.77**	1.75**	1.74**
Self-reported protozoa	0.84	0.82	0.84
Adult age (15 or older)	7.75**	7.47**	7.97**
Sex as female	0.64*	0.64*	0.63*
Rural Residence	1.97**	1.91**	1.99**

==

Note: ‘A’ stands for antibody result for schistosomiasis. ‘SR’ is self report for schistomiasis. **p<0.05.
